# Aquaporin 5 Expression in Mouse Mammary Gland Cells Is Not Driven by Promoter Methylation

**DOI:** 10.1155/2015/460598

**Published:** 2015-02-12

**Authors:** Barbara Arbeithuber, Roland Thuenauer, Yasmin Gravogl, Zsolt Balogi, Winfried Römer, Alois Sonnleitner, Irene Tiemann-Boege

**Affiliations:** ^1^Center for Advanced Bioanalysis GmbH, 4020 Linz, Austria; ^2^Institute of Biophysics, Johannes Kepler University, 4020 Linz, Austria; ^3^Institute of Biology II, Albert Ludwigs University of Freiburg, 79104 Freiburg, Germany; ^4^BIOSS Centre for Biological Signalling Studies, Albert Ludwigs University of Freiburg, 79104 Freiburg, Germany; ^5^Weill Institute for Cell & Molecular Biology, Department of Molecular Biology & Genetics, Cornell University, Ithaca, NY 14853, USA

## Abstract

Several studies have revealed that aquaporins play a role in tumor progression and invasion. In breast carcinomas, high levels of aquaporin 5 (AQP5), a membrane protein involved in water transport, have been linked to increased cell proliferation and migration, thus facilitating tumor progression. Despite the potential role of AQP5 in mammary oncogenesis, the mechanisms controlling mammary AQP5 expression are poorly understood. In other tissues, AQP5 expression has been correlated with its promoter methylation, yet, very little is known about AQP5 promoter methylation in the mammary gland. In this work, we used the mouse mammary gland cell line EpH4, in which we controlled AQP5 expression via the steroid hormone dexamethasone (Dex) to further investigate mechanisms regulating AQP5 expression. In this system, we observed a rapid drop of AQP5 mRNA levels with a delay of several hours in AQP5 protein, suggesting transcriptional control of AQP5 levels. Yet, AQP5 expression was independent of its promoter methylation, or to the presence of negative glucocorticoid receptor elements (nGREs) in its imminent promoter region, but was rather influenced by the cell proliferative state or cell density. We conclude that AQP5 promoter methylation is not a universal mechanism for AQP5 regulation and varies on cell and tissue type.

## 1. Introduction

Aquaporin 5 (AQP5) is a highly conserved transmembrane channel formed by four subunits, which passively transports water in and out of cells according to the osmotic gradient across the membrane (reviewed in [1, 2]). The expression of AQP5 is tissue specific and tightly regulated, with high expression levels in lung, salivary glands, and lachrymal tissue. In the mammary gland, AQP5 expression varies throughout different stages of mammary tissue differentiation. AQP5 is only expressed in ductal epithelial cells during virgin development, but is absent during pregnancy and after parturition in mice [3]. Similar observations were made in rats, which had a weak mRNA and no detectable AQP5 protein expression in the mammary gland during lactation [4]. Interestingly, mammary tumor libraries showed increased AQP5 mRNA levels, whereas mRNA libraries of normal mammary glands of lactating mice showed low levels [3].

Recent studies have revealed that aquaporins likely play a role in tumor progression and invasion, with altered expression observed in several tumor types [5–9]. AQP5 is highly expressed in metastasized colon cancer tissue and was associated with cell proliferation and metastasis of colon cancer cells to the liver [5]. Increased AQP5 expression was also observed in non-small cell lung cancer [6, 10]. Lung cancer cells with high AQP5 expression had enhanced proliferation and migration potential, while cells with reduced AQP5 expression had low metastatic activity [6]. It was also shown that in benign tumor and invasive carcinoma, there is a change of AQP5 expression related to the breast cancer grade [7]. Moreover, reduction of AQP5 expression, achieved by increased osmotic stress or an inhibitory RNA, was associated with a significant reduction in cell proliferation and migration in the breast cancer cell line MCF-7 [7]. Based on these observations, it has been suggested that AQP5 plays a role in cell growth and metastasis in human breast cancer [7]. Thus, a better understanding of the factors that affect AQP5 expression in the mammary gland might lead to a better insight into the oncogenic activity of this tissue and potentially to novel antibreast cancer therapies.

The mechanisms controlling AQP5 expression are not very well understood, but expression of AQP5 has been correlated with methylation levels of its promoter, with a reduced expression when the promoter was highly methylated [11–13]. The methylation of the putative Sp1 binding sites (Sp1-1, Sp1-2, and Sp1-3) for the transcription factor specificity protein 1 (Sp1) especially reduced AQP5 expression [11, 12]. In a human salivary gland ductal cell line that does not constitutively express AQP5, the expression of AQP5 was induced by demethylation of specific CpG sites within the region of Sp1 binding sites. Moreover, the effect of demethylation of several sites was additive [11]. Additionally, in cultured rat alveolar epithelial cells a decrease in methylation of the AQP5 promoter region was associated with an increase in Sp1 binding and AQP5 expression [12]. In a different study, treatment of a murine aging model with a global DNA demethylating agent (5-Aza 2′ deoxycytidine) lead to an increased volume of salivary flow, which was coupled to an increase in AQP5 expression [13]. Therefore, it was proposed to restore hyposalivation for age-related xerostomia using DNA demethylating agents as a potential drug.

In this work, we analyzed the role of promoter methylation in the regulation of AQP5 expression in EpH4 mammary epithelial cells. EpH4 cells are nontumorigenic cells derived from spontaneously immortalized mouse mammary epithelial cells [14], which can be used as a mammary gland model system, since the initial stages of mammary gland differentiation can be mimicked* in vitro* [15] by treating EpH4 cells with the steroid hormone dexamethasone (Dex). Dex is a synthetic steroid hormone of the glucocorticoid group, shown in an* in vitro* mammary gland system, to induce milk production when supplied in a lactogenic mix, mimicking lactation* in vivo* [15]. AQP5 expression and promoter methylation were monitored in both, dividing and nondividing EpH4 cells treated with Dex. Treatments with Dex resulted in reduced levels of AQP5, as it normally occurs with AQP5 during lactation* in vivo* [4]. The expression of mRNA was reduced before measurable changes in AQP5 protein were detected, suggesting that AQP5 downregulation in our system is likely controlled at the transcriptional level. Yet, AQP5 transcription was not associated with measurable changes of AQP5 promoter methylation in our system nor with negative glucocorticoid response elements (nGREs), found ubiquitously in the AQP5 promoter. In contrast, the division state of the cell was found to be relevant in the regulation of AQP5, and AQP5 expression was effectively downregulated in nondividing or confluent cells, but not in actively dividing cells, suggesting that the proliferative state of the cells or the cell density plays an important role in the response to mammary AQP5 regulation. Finally, our observations preclude the use of methylation as a regulator for mammary gland AQP5 expression.

## 2. Materials and Methods

### 2.1. Cell Culturing and Reagent Treatments

EpH4 cells [14] were cultured in Dulbecco's Modified Eagle Medium (DMEM) containing 5% heat inactivated fetal calf serum (FCS) supplemented with 2 mM L-Glutamine. Cells were routinely dispensed on plastic tissue-culture dishes at a density of about 70% confluence and cultured in 5% CO_2_ at 37°C and 95% humidity as described previously [14, 16]. Washes were performed using phosphate buffered saline (PBS) (without CaCl_2_; MgCl_2_). For all the experiments, cells were seeded and cultured in 6-well cell culture plates. All cell culturing reagents were obtained from Gibco.

#### 2.1.1. “Dividing” Culturing Conditions

Cells were seeded from the same original stock and grown to approximately 70% confluence and then treated with 0.1 *μ*M dexamethasone as used previously [15] or 1 *μ*M 5-Aza 2′ deoxycytidine (5-Aza) (Sigma). A viable 5-Aza concentration was first tested given the strong global effect of this chemical on overall transcription (see Supplementary Figure 1 in Supplementary Material available online at http://dx.doi.org/10.1155/2015/460598).

#### 2.1.2. “Nondividing (Confluent)” Culturing Conditions

Cells were grown to 100% confluence and washed with PBS twice. Then the medium was exchanged to DMEM supplemented with L-Glutamine, 0.2% bovine serum albumin (BSA), and 0.1 *μ*M Dex.

#### 2.1.3. “Nondividing (Matrigel-Treated)” Culturing Conditions

Cells were grown to 80%–90% confluence and washed with PBS twice. Then the medium was exchanged to DMEM supplemented with L-Glutamine and 0.2 mg/mL Matrigel (BD Biosciences). Matrigel culturing conditions rendered easily reproducible cell numbers and overall expression levels of total RNA. Matrigel was always handled at 4°C and warmed to room temperature before addition to the cells. After 24 h, the cells were treated with 0.1 *μ*M Dex or 5 *μ*M 5-Aza in DMEM supplemented with L-Glutamine.

Cell numbers were determined with a Z2 Coulter Particle Count and Size Analyzer (Beckman Coulter). In order to ensure that EpH4 cells had the same age throughout different experiments and treatment times at the analysis time point, if not stated otherwise, the longest treatment was performed first followed by other treatments to corresponding time points relative to the extraction.

### 2.2. Preparation of Cell Lysates

Cultured cells were washed with 2 mL PBS twice and then trypsinized (using 500 *μ*L trypsin) for 5 to 25 minutes until cells detached from the dish surface. Duration of trypsinization depended on the cell culturing conditions; cells treated with Matrigel needed longer trypsinization time. To remove trypsin after detachment, cells were washed with PBS, centrifuged, and resuspended in 100 *μ*L PBS per well resulting in cell numbers of approximately 10^5^ to 10^7^ cells/mL. If more than 100 *μ*L cell suspension was needed, several wells of the same treatment condition were pooled. The aliquot of 100 *μ*L of cell suspension was pelleted, followed by the removal of the supernatant and resuspension of the cell pellet in 100 *μ*L RIPA buffer (50 mM Tris-HCl, pH 7.4, 150 mM NaCI, 2 mM EDTA, 1% Triton X-100, and 1% sodium-deoxycholate), supplemented with protease inhibitors (4 *μ*g/mL Leupeptin, 500 *μ*M PMSF, and 4 *μ*g/mL Aprotinin (Sigma)). Cells were incubated on ice for 20 minutes and vortexed every few minutes. After lysis, the cell debris was removed by centrifugation. Protein concentration was determined with the Bradford assay.

### 2.3. Analysis of AQP5 Protein Expression by Western Blotting

Approximately 30 *μ*g of total protein from the cell lysate was loaded per lane on a 12% SDS-PAGE gel, electrophoresed, and blotted on a polyvinylidene fluoride (PVDF) membrane (Millipore). Unspecific binding sites were blocked by incubating the membrane with 5% nonfat dry milk (BioRad) in PBS containing 0.1% Tween 20 (PBS-T). A primary rabbit anti-AQP5 antibody (Alomone labs; dilution 1 : 500) and a secondary anti-rabbit HRP-conjugated antibody (Amersham GE Healthcare; dilution 1 : 1000) was used to measure levels of AQP5 protein. Beta-tubulin (TUBB) was used as loading control. TUBB was detected by a primary mouse monoclonal anti-beta-tubulin antibody (Sigma; dilution 1 : 1000) and a secondary anti-mouse HRP-conjugated antibody (GE Healthcare; dilution 1 : 1000). Blots were incubated with ECL Western Blotting Detection Reagents (GE Healthcare) and then developed on an Amersham Hyperfilm ECL (GE Healthcare).

### 2.4. Analysis of AQP5 Protein Expression by Flow Cytometry

For fluorescence activated cell sorting (FACS) measurements, cells were cultured and trypsinized as described before. Trypsinization was carried out until the culture was broken into single cells. About 4 × 10^6^ cells were fixed with 1% formaldehyde in PBS (with CaCl_2_ and MgCl_2_) for 15 min at room temperature (RT) and centrifuged at 480 g for 5 min. Permeabilization was performed by incubating cells with 0.2% Triton X-100 for 5 min followed by centrifugation and blocking with 1% BSA in PBS and centrifugation again. Cells were incubated at RT for 30 min with primary anti-AQP5 antibody and then with a secondary anti-rabbit Cy5-conjugated antibody (GE Healthcare). Labeled cells were washed with PBS twice and diluted in 200 *μ*L PBS. Levels of AQP5 per cell were measured with a BD FACSAria instrument (BD Biosciences). Control experiments were performed without primary antibody to establish the background fluorescence. Data was normalized to tubulin and shown relative to the untreated sample.

### 2.5. Extraction of mRNA and Analysis with qPCR

Total RNA was extracted from a 100 *μ*L trypsinized EpH4 cell suspension with the ZR RNA MiniPrep Kit (Zymo Research). All steps were performed according to manufacturer's instructions. The cDNA synthesis was performed with the Phusion RT-PCR Kit (Finnzymes) according to manufacturer's instructions. Approximately 1 *μ*g RNA was converted into cDNA. We either performed a two-step quantitative PCR (qPCR) with the SensiMix SYBR No-ROX Kit or the one-step qPCR with the SensiMix SYBR No-ROX One-step Kit (both from Bioline). Glyceraldehyde 3-phosphate-dehydrogenase (GAPDH) was used as a housekeeping gene for normalization purposes of the total RNA versus the AQP5 RNA. The qPCR reactions were carried out with a reverse transcription step at 42°C for 10 min or directly starting with an initial heating step of 95°C for 10 min followed by 40 cycles at 95°C for 15 sec, 57°C for 15 sec, and 72°C for 15 sec in a LightCycler 480 (Roche) or a CFX96 Real-Time PCR Detection System (BioRad). The primer concentration for qAQP5 and qGAPDH was 0.5 *μ*M. Primers were designed to span exon-exon junctions, ensuring that only cDNA and not genomic DNA was amplified. Primer sequences used throughout this work are shown in Supplementary Table 1.

Plasmids with an AQP5 and GAPDH insert were used as standards with known concentrations (10^7^, 10^6^, 10^5^, 10^4^, and 10^3^ molecules/reaction) to normalize AQP5 and GADPH for interexperimental variation of qPCR. Plasmids were prepared from AQP5 and GAPDH PCR products, cloned with the InsTAclone PCR Cloning Kit (Fermentas), and purified with the GenElute Plasmid Midiprep Kit (Sigma). Each insert was verified by sequencing.

### 2.6. Analysis of DNA Methylation

Bisulfite treatment was performed with the EZ DNA Methylation-Direct Kit (Zymo Research) on trypsinized cells according to manufacturer's instructions. Bisulfite treatment was performed either starting with whole cells or purified DNA. 10 *μ*L cell suspension (which equals 10^4^-10^5^ cells) or 400 ng genomic DNA was used for bisulfite treatment. No difference in conversion efficiency was observed between whole cells or with purified DNA. Bisulfite treated DNA was amplified with 1x ZymoPreMix, 0.5 *μ*M forward and 0.5 *μ*M reverse m-1 primer, Zymo*Taq* DNA Polymerase (Zymo Research) cycled for 35 times at 95°C for 30 sec, 57°C for 35 sec, and 72°C for 45 sec with an initial denaturation step of 95°C for 10 min; and a final extension at 72°C for 7 min. The conversion efficiency was monitored by amplifying 20 ng of bisulfite treated DNA with 0.5 *μ*M primers specific for unconverted DNA (uc-3) denatured at 95°C for 10 min and cycled for 32 times at 95°C for 30 sec, 57°C for 35 sec, and 72°C for 45 sec with a final extension at 72°C for 7 min 1x ZymoPreMix and Zymo*Taq* DNA Polymerase (Zymo Research). We analyzed 20 *μ*L of the reaction on a 10% polyacrylamide gel together with a standard series amplified from 20 ng, 10 ng, 5 ng, and 2 ng unconverted DNA. We performed the same experiment with qPCR using the SensiMix SYBR No-ROX Kit and a standard series of 25 ng, 5 ng, 1 ng, and 0.2 ng extracted unconverted DNA.

#### 2.6.1. TA Cloning and Sequencing

Amplified bisulfite treated DNA (20 *μ*L) was gel purified on a 1% agarose-gel and the QIAquick Gel-extraction Kit (QIAGEN). DNA was eluted in 30 *μ*L deionized H_2_O and ligated into the pTZ57R/T vector using the InsTAclone PCR Cloning Kit (Fermentas), using an insert to vector ratio of 10 to 1. Ligation products were transformed into NEB 5alpha competent* E. coli* (High Efficiency) (New England Biolabs) and plated on ampicillin LB plates. Plasmids positive for an insert were screened and prepared for sequencing with the ZR Plasmid Miniprep Classic Kit (Zymo Research) or the QIAprep Spin Miniprep Kit (QIAGEN) and sequenced by Beckman Coulter Genomics or Microsynth.

Restriction analysis of DNA methylation was performed by digesting amplified DNA (primer: m-1, 0.5 *μ*M) for 1 h with 1 unit/reaction Fnu4HI or BssH4 (New England Biolabs). Fragments were separated on a 10% polyacrylamide gel.

### 2.7. Bead-Emulsion Amplification (BEA)

Bisulfite treated DNA was amplified as described in the section of analysis of DNA methylation. A 1 : 40,000 dilution of the PCR product was analyzed using bead-emulsion amplification (BEA) following conditions as described previously [17, 18] with a few changes. Specifically, the PCR product was hybridized to magnetic beads covered with R-CSX primer at 94°C for 2 min, followed by 58°C for 15 min with repeated stirring, and kept at 72°C until further use. For the aqueous phase we used 10x Titanium buffer, 8 mM MgCl_2_, 1 mM dNTP mix, 9.3 *μ*M RBGN-tag primer or REV primer, 0.05 *μ*M FWD primer, and 2 *μ*L Titanium Taq polymerase (Clontech) in a total volume of 150 *μ*L. The aqueous phase was emulsified with 600 *μ*L oil using a homogenizer PowerGen 125 (Fisher Scientific) at speed 4.5 for 100 sec. Emulsion reactions were amplified at 94°C for 2 min followed by 55 cycles at 94°C for 15 sec, 54°C for 30 sec, and 68°C for 75 sec and a final extension step at 72°C for 5 min. The beads were labeled with probes AQP5-CpG8 (C) and AQP5-CpG8 (T) using the following temperatures: 94°C for 2 min, 52°C for 5 min, and 60°C for 5 min. The beads were held at 72°C until stripped with 1E buffer as described previously [17, 18].

### 2.8. Promoter Transfection Experiments with Luciferase Vector

A construct of the murine AQP5 promoter (2021 bp upstream of the ATG start codon) was produced by the fusion of three overlapping fragments treated with restriction enzymes followed by ligation. Two fragments were synthesized (gBlock fragments-IDT), reaching from position chr15: 99589250-99589987 (gBlock1) and chr15: 99589970-99590606 (gBlock2) of the GRCm38/mm10 assembly and the third was obtained by PCR of region Chr15:99590527-99591271. The amplicon was produced with genomic DNA from EpH4 cells extracted using the Gentra Puregene Cell Kit (QIAGEN) according to manufacturer's instructions and amplified using 0.125 U OneTaq Hot Start DNA polymerase (NEB) with an initial heating step of 94°C for 5 min followed by 45 cycles at 94°C for 15 sec, 58°C for 15 sec, and 68°C for 45 sec (primer sequences are shown in Supplementary Table  1).

After digestion of the three DNA fragments and the target vector (pGL3-basic, Promega) using the respective restriction enzymes (gBlock1:* Xho*I and* Pst*I, gBlock2:* Pst*I and* Pvu*I, PCR amplicon 3:* Pvu*I and* Hind*III, pGL3-basic:* Xho*I and* Hind*III, NEB, 90 min 37°C) the DNA fragments and the vector were purified using the Wizard SV Gel and PCR Clean-Up System (Promega) according manufacturer's instructions. The inserts and the vector were ligated in a 1 : 3 molar ratio using the T4 DNA ligase (NEB) in an overnight incubation at 16°C. Half of the ligation reaction (10 *μ*L) was transformed into XL1-Blue competent* E. coli* cells (Agilent) according to manufacturer's instructions and plated on LB-agar containing 100 *μ*g/mL ampicillin. Screening for positive clones was performed by colony PCR and control restriction digests. The integrity of the final pGL3-construct containing the AQP5 promoter upstream of the* luc*+ gene (Firefly) was verified by sequencing. A single colony of the positive clone was inoculated in 3 mL LB medium containing 100 *μ*g/mL ampicillin and an overnight culture was grown, shaking at 37°C. 2 mL of culture was harvested by centrifugation and a plasmid Miniprep was performed using the PureYield Plasmid Miniprep System (Promega) according to manufacturer's instructions.

The same purification procedure was used to obtain high amounts of (promoter-less) pGL3-basic vector (Promega) and the pRL-TK vector (Promega), as well as the peGFP-N1 vector (Clontech) for transfection experiments.

Transient transfections of EpH4 cells were performed in 10 cm tissue-culture dishes (Sarstedt) at 80–90% confluence using Lipofectamine 2000 (Life Technologies) according to manufacturer's instructions. Briefly, 24–40 *μ*g of total plasmid DNA (luciferase reporter gene vectors) were used to prepare a DNA-transfection reagent mixture with Opti-MEM reduced serum medium (Life Technologies), and 60 *μ*L Lipofectamine 2000. The reagent mixture contained the pGL3-basic vector with the AQP5 promoter region in a 1 : 1 ration with the pRL-TK (R*luc*/TK, Promega) plasmid, which expresses Renilla luciferase, to normalize for transfection efficiency. A promoter-less (empty) pGL3-basic vector was used as an expression control. After 20 min incubation at room temperature, the mixture was added drop by drop to the cells with or without 0.1 *μ*M Dex. Dex was added 4 h prior to the transfection agents in one set of experiments. In the other set, Dex treatments were performed simultaneously with the transfection and again 4 h after transfection, as well as, 2 h prior to harvesting the cells for downstream measurements. The medium was changed to DMEM supplemented with L-Glutamine and 0.2 mg/mL Matrigel 4 h after transfection. Then, 38 h after transfection, cells were harvested and processed to measure the luciferase activity and the levels of AQP5,* Firefly* luciferase (*luc*+) and* Renilla *luciferase (R*luc*). Cells harvested from a 10 cm dish by trypsinization were resuspended in 200 *μ*L PBS and split for the luciferase assay and for the mRNA extraction.

Luciferase activity was measured by a luminometer (TECAN infinite M200 pro multiwell reader) using the Dual-Glo Luciferase Assay System (Promega). After harvesting, the cells were transferred in a 96-well plate (Greiner, black, clear bottom). The well-bottoms were covered with a blank (white) sheet of paper to avoid cross talk between wells. 100 *μ*L Dual-Glo Luciferase Assay Reagent (Promega) were added to the cells and* Firefly* activity was measured after 10 min and 15 min incubation at room temperature. Subsequently, 100 *μ*L Dual-Glo Stop and GloReagent were added and* Renilla* activity was measured after 10 min and 15 min incubation at room temperature.

In order to monitor the response of Dex the transfected cells, we also monitored AQP5 and GAPDH expression at the mRNA level: total RNA was extracted from a 100 *μ*L trypsinized EpH4 cell suspension with the Quick-RNA MiniPrep Kit (Zymo Research). All steps were performed according to manufacturer's instructions. Expression changes were monitored as described above (*Extraction of mRNA and analysis with qPCR*), except that the SensiMix SYBR No-ROX One-step Kit (Bioline) was used for onestep qPCR.

### 2.9. AQP5 Expression in Human Mammary Gland MCF-7 Cells

MCF-7 cells, obtained from the cell repository of the University of Freiburg, Germany, were cultured in DMEM containing 10% FCS at 5% CO_2_ at 37°C and 95% humidity. Cells were seeded on 24 well plates and cultured for 2 days (yielding subconfluent cell layers with approximately 50% confluence) or 5 days (yielding confluent cell layers). After stimulation with Dex for different time periods, the cells were washed with PBS, trypsinized, and resuspended in DMEM. The pellet was washed two times with PBS before mRNA extraction with the Quick-RNA MiniPrep Kit (Zymo Research); cDNA synthesis was performed with the ProtoScript II Reverse Transcriptase (NEB), both according to manufacturer's instructions. AQP5 and GAPDH expression were quantified by qPCR with the qAQP5_human and qGAPDH_human primers, respectively, using 0.25 U Phusion HotStart II DNA Polymerase (Thermo Scientific) with an initial heating step of 98°C for 30 sec followed by 45 cycles at 99°C for 5 sec, 63°C for 15 sec, and 72°C for 5 sec (see primer sequences in Supplementary Table  1). Primers were designed to anneal in conserved regions between humans and mice; therefore, the same plasmids could be used as standards (10^7^, 10^6^, 10^5^, 10^4^, and 10^3^ molecules/reaction) for measuring murine or human absolute AQP5 mRNA levels.

### 2.10. Statistical and Computational Data Analysis

Values are presented as means ± standard errors. Statistical comparisons were performed with the two-tailed Student's *t*-test for unpaired samples (when the number of groups was 2). A value of *P* < 0.05 was considered to be significant and indicated by asterisks in the figures as ^*^ *P* < 0.05, ^**^ *P* < 0.005.

## 3. Results

### 3.1. Dex Reduces AQP5 Expression in a Nondividing Mouse Mammary Cell System

**Figure 1:**
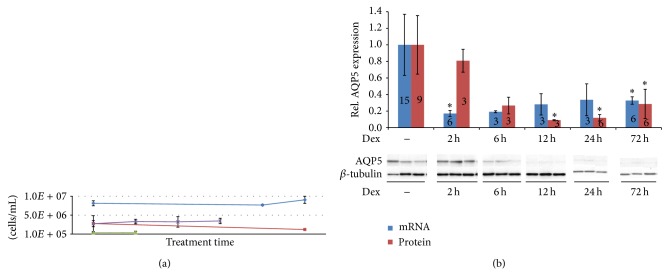
Analysis of AQP5 expression in nondividing EpH4 cells treated with Matrigel. (a) Shown is the average concentration of trypsinized cells [cells/mL] counted with a Coulter Counter from 4 different experiments. Averages are represented as crosses with confidence intervals; *n* = 3; alpha = 0.05. Treatments were performed such that the age of the cells from seeding to the time of extraction was the same within one experiment. (b) Levels of AQP5 mRNA or protein measured after shorter or longer Dex treatment times. Cells were treated with Dex for 2 h, 6 h, 12 h, 24 h, and 72 h. Numbers inside bars represent experimental replicates; mRNA was normalized to GAPDH; protein was normalized to TUBB and shown relative to untreated samples. Bars represent averages and confidence intervals; *n* = 3–15; alpha = 0.05. Significance was tested between controls (−Dex) and different Dex treatments, with asterisks denoting *P* < 0.05.

We analyzed the effect of the steroid hormone dexamethasone (Dex) on AQP5 expression in epithelial mammary mouse cells, EpH4, at the mRNA and protein level. A solubilized basement membrane matrix, Matrigel, was used to culture EpH4 cells, which stimulates cells to form an* in vitro* mammary gland differentiation system, as reported previously [15]. Matrigel is secreted by Engelbreth-Holm-Swarm mouse sarcoma cells and forms a gelatinous protein mixture that resembles the extracellular environment found in many tissues* in vivo* [19, 20]. We observed that cells stopped dividing when Matrigel was added to ~80% confluent EpH4 cells for 24 h (Figure 1(a)). Incubation of these nondividing EpH4 cells with Dex resulted in a 4-6-fold reduction of AQP5 expression at the mRNA and protein level measured at different time points of a maximal 72-hour time frame (Figure 1(b)). Downregulation of AQP5 was effective even with Dex treatments of a week (Supplementary Figure  2). AQP5 downregulation is a direct effect of Dex treatment and not a response to the components of Matrigel, since no reduction in AQP5 expression was observed in control cells, to which no Dex was added after incubation with Matrigel for 24 hours (Figure 1). Interestingly, nondividing EpH4 cells that were not treated with Matrigel but were 90–100% confluent and stopped dividing also showed a decrease of AQP5 protein levels when incubated with Dex (Supplementary Figure  3). These results show that (1) AQP5 expression can be reduced with Dex in nondividing mammary mouse cells; (2) Matrigel is not necessary to achieve this downregulation.

We also tested how fast Dex treatments affected AQP5 expression. After 2 hours of Dex treatment, a significant reduction was already observed for AQP5 mRNA but not for AQP5 protein (Figure 1(b)). Both mRNA and protein levels were downregulated after 6 hours of treatment. Our results show that AQP5 mRNA is downregulated before protein levels decrease and indicate that AQP5 protein levels are likely linked to changes in mRNA levels.

The observed downregulation of AQP5 by Dex is not due to changes in cell numbers or viability, since the number of viable cells stayed constant after Dex treatments (Figure 1(a)). We also examined if AQP5 expression was similar in all cells by measuring the AQP5 protein levels in individual cells with FACS. These experiments showed that 80 to 90% of the cells had similar AQP5 expression levels, which shifted as a whole upon Dex treatment, and demonstrate that the AQP5 expression of the whole cell population was reduced by Dex (Supplementary Figures  4 and 5). Moreover, changes in AQP5 protein expression after 24 and 72 hours of Dex treatment measured by FACS were comparable to changes measured in Western blots (Supplementary Figure 5).

### 3.2. The Proliferative Activity of Cells Affects the Response to Dex

**Figure 2:**
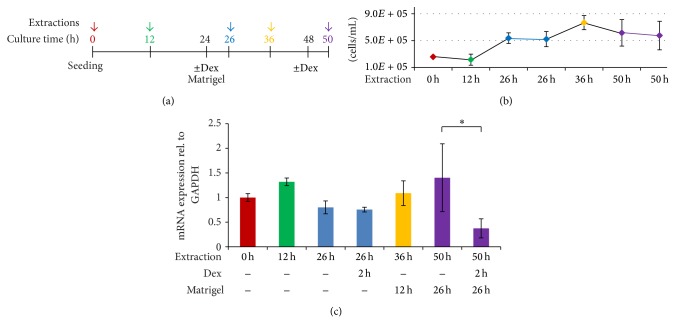
Cell growth and AQP5 expression at different cell culturing stages. (a) Cell culturing and treatment times at which cells were harvested (indicated with arrows). (b) Numbers of cultured cells were counted with a Coulter Counter. Shown are averages (dots) and confidence intervals; *n* = 3; alpha = 0.05. (c) Total mRNA was extracted from cells used for seeding, 12 h after seeding, 26 h after seeding (with or without 2 h Dex treatment), 36 h after seeding with 12 h Matrigel treatment, and 50 h after seeding with 26 h Matrigel treatment (with or without 2 h Dex treatment). AQP5 expression is normalized to GAPDH and shown relative to the 0 time point treatment. Bars represent averages and confidence intervals; *n* = 3; alpha = 0.05. Significance was tested between the 26 h treatments (−Dex, −Matrigel, 26 h and 2 h Dex, −Matrigel, 26 h) and between the 50 h treatments (−Dex, 26 h Matrigel, 50 h and 2 h Dex, 26 h Matrigel, 50 h).

The level of AQP5 mRNA and number of viable cells were monitored at several culturing times, from actively dividing conditions up to the nondividing state reached after Matrigel treatments. As shown in Figure 2, cell numbers doubled during the first 24 hours after sparse seeding of EpH4 cells. The addition of Dex at this stage did not result in a significant reduction of AQP5 mRNA, contrary to our observations in nondividing cells. When Matrigel was added to the same cell culture, the number of cells increased ~25% within the first 12 hours and then remained constant. The incubation with Dex for two hours after 24 hours of Matrigel treatment resulted again in a 4-6-fold AQP5 downregulation.

In a separate experiment, 12 cell culture replicates were seeded at subconfluent density and treated with Dex at ~70% confluence for 2 hours or 24 hours. The expression of AQP5 mRNA was significantly reduced with Dex treatments in 12 replicates, but the reduction was only twofold and not as strong as observed in polarized cells treated with Matrigel or 90–100% confluent cells (Supplementary Figure 6).  Table 1 shows a summary of AQP5 regulation upon Dex treatment for different EpH4 culturing conditions. In actively dividing cells, Dex induced only a weak (0-2-fold) decrease in AQP5 mRNA and protein levels. In nondividing or confluent cells, Dex induced a stronger (4-6-fold) downregulation of AQP5 mRNA and protein levels. These results show that the Dex-mediated downregulation of AQP5 is influenced by the proliferative state of EpH4 cells.

### 3.3. Does Promoter Methylation Influence the Dex Response?

**Figure 3:**
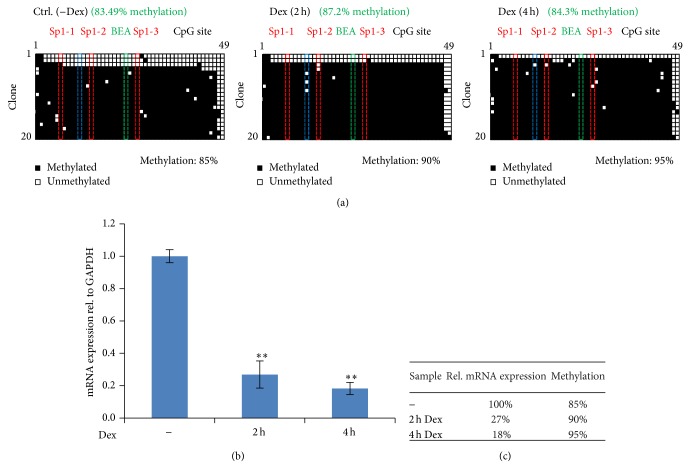
Analysis of AQP5 promoter methylation. (a) DNA methylation of 49 CpG sites found in the 289 base pair region located ~70 bases from the start codon of the AQP5 promoter, measured by bisulfite sequencing of individual clones (total methylation percentage shown in black). A single CpG site (CpG#24) was measured in tens of thousands of molecules with bead-emulsion amplification (methylation percentage shown in green). Nondividing cells without Dex and with Dex treatments for 2 h and 4 h were analyzed. (b) Changes in AQP5 mRNA expression analyzed with one-step qPCR. Total mRNA was extracted from Matrigel-treated cells without Dex and with Dex treatments for 2 h and 4 h. AQP5 expression is normalized to GAPDH and shown relative to no Dex treatment. Bars represent averages and confidence intervals; *n* = 3; alpha = 0.05. Significance was tested between control (−Dex) and differently treated samples. (c) Summary of AQP5 mRNA expression and promoter methylation.

Since expression of AQP5 can be downregulated by high methylation levels of its promoter [11–13], we investigated if methylation of the AQP5 promoter played a role in the observed reduction of AQP5 expression. We analyzed the methylation levels of a 289 base pair region located ~70 bases from the start codon of the minus strand of the AQP5 promoter containing 49 CpG sites (Supplementary Figure 7). This region contains three putative Sp1 binding sites (Sp1-1, Sp1-2, and Sp1-3) that could be potential targets of regulation by methylation [11, 12, 21–23]. We analyzed the methylation levels by sequencing up to 20 individual bacterial clones containing bisulfite converted DNA. No notable changes in methylation levels were observed among different Dex treatments, although AQP5 expression was reduced by ~5-fold (Figure 3). AQP5 expressing cells showed as much methylation as cells with reduced AQP5 levels. Furthermore, none of the three Sp1 binding sites showed consistent differences in methylation. Interestingly, individual molecules were either fully methylated or unmethylated throughout most of the 49 analyzed CpGs.

In order to detect very small differences in methylation, a single CpG was analyzed using bead-emulsion amplification (BEA) in nondividing, Matrigel-treated EpH4 cells. With this method, tens of thousands single DNA molecules were analyzed in parallel for CpG#24. The BEA results confirmed that 84–88% of the cells have methylated DNA (Supplementary Table 2) regardless of Dex treatment or levels of AQP5 expression. Similar results were obtained with restriction fragment length polymorphism analysis of bisulfite converted DNA, in which 4 different CpG sites were targeted using BssHII, that specifically recognizes bisulfite converted, methylated DNA (Supplementary Figure 8). In a separate experiment, we also assessed the methylation levels in dividing EpH4 cells, grown at ~70% confluence (12 replicates). Based on the analysis of 26 to 31 clones derived from bisulfite converted DNA, the methylation ranged between 73 and 89% throughout all 49 CpG sites of the AQP5 promoter and was again not correlated with levels of AQP5 expression (Supplementary Figure 6).

**Figure 4:**
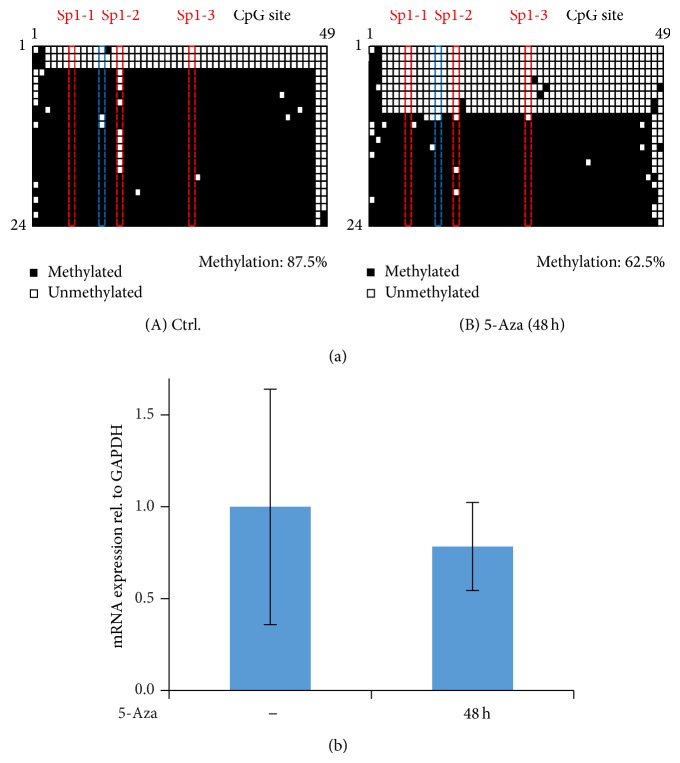
Changes in DNA methylation with the demethylating agent 5-Aza in actively dividing cells. (a) Changes in DNA methylation monitored by bacterial bisulfite sequencing of individual clones. Cells were treated with 5-Aza for 48 h while still dividing. Data for the control (no Aza) are combined from two experiments, one treated with Matrigel (also shown in Supplementary Figure 9, treatment 1) and one without Matrigel. (b) Expression of AQP5 mRNA analyzed with qPCR. AQP5 expression is normalized to GAPDH and shown relative to the no-5-Aza treatment. Bars represent averages and confidence intervals; *n* = 6; alpha = 0.05. Significance was tested between control (5-Aza) and the 5-Aza treated sample.

We further assessed the effect of 5-Aza, a global inhibitor of DNA methylation, in order to validate our DNA methylation detection methods. It is expected that 5-Aza has the strongest effect in replicating cells, when it gets incorporated as an analogue of deoxycytidine. 5-Aza irreversibly binds and depletes DNA methyltransferases (DNMTs) that reestablish methylation marks on hemimethylated DNA during cell division (reviewed in [24, 25]). We treated actively dividing cells with 5-Aza, 48 h before reaching 70% confluence, and monitored the changes in DNA methylation. The 5-Aza concentration was standardized* a priori* by a viability test, given the strong global effect of this chemical on overall transcription (Supplementary Figure 1). Our results show that a 48-hour incubation time with 1 *μ*M 5-Aza caused a notable AQP5 promoter demethylation, from 87.5% to 62.5% (Figure 4). Interestingly, levels of AQP5 mRNA were not affected by 5-Aza treatments, despite the notable changes in AQP5 promoter methylation.

As expected, incubation of nondividing, Matrigel-treated EpH4 cells with 5-Aza did not influence the levels of DNA methylation nor AQP5 expression (Supplementary Figure 9). These results show that (1) our methodology can detect differences in DNA methylation; (2) 5-Aza causes demethylation when cells are replicating, but not when they are quiescent and polarized in Matrigel; and (3) AQP5 promoter methylation does not influence the changes in AQP5 expression.

### 3.4. Role of Other Elements in the AQP5 Promoter

**Figure 5:**
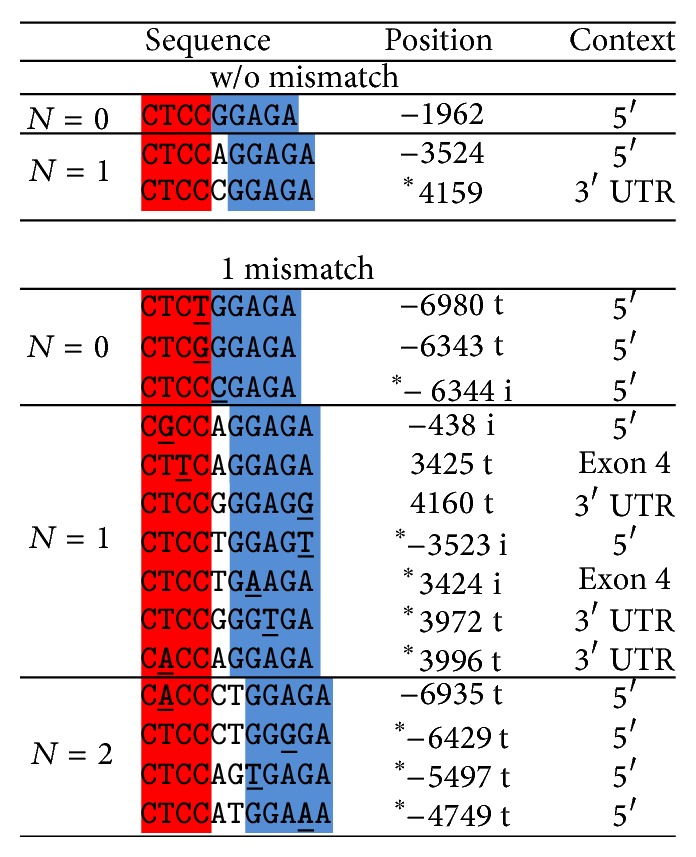
nGRE motifs in the AQP5 promoter region. The ~7 kb region of the AQP5 promoter and 4.5 kb of the AQP5 gene were analyzed for nGRE motifs with 0, 1, or 2 nucleotides (*N*) as a spacer with and without mismatches. Sequence positions are calculated relative to the translation initiation site. For the motifs containing a mismatch, the mismatch was classified as tolerable (t) or intolerable (i) for glucocorticoid binding according to Surjit et al. [26]. Mismatches are indicated bold and underlined.

Alternative to methylation, AQP5 expression could be directly affected via several regulatory elements such as the negative glucocorticoid response elements (nGREs) [26] that could mediate AQP5 repression induced by Dex. Thus, we examined whether the AQP5 promoter accounts for the observed downregulation of AQP5 by Dex in transfection experiments. We transfected EpH4 cells with a luciferase reporter vector (Firefly) containing 2021 bp of the upstream region from the translation initiation site of the murine AQP5, which includes two nGRE motifs at position −438 and at position −1962 (Figure 5).

**Figure 6:**
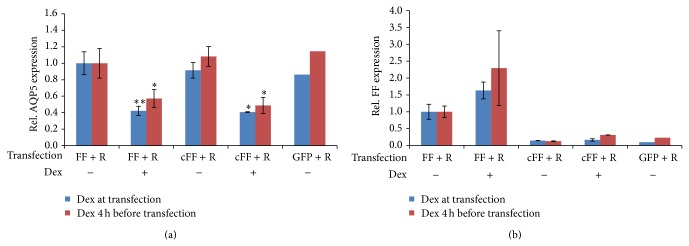
Effect of Dex on the AQP5 promoter. EpH4 cells were treated with Dex, 4 h prior (*n* = 4) or during transfection (*n* = 5) of the luciferase expression pGL3-basic vector including the AQP5 promoter (FF) and the pRL-TK transfection control plasmid (R). Negative controls included the pGL3-basic vector without promoter (cFF) (*n* = 2) and the peGFP-N1 vector (GFP), (*n* = 1). (a) Levels of AQP5 mRNA normalized to GAPDH are shown for the different transfected EpH4 cells with or without Dex treatment. Bars represent averages and confidence intervals; alpha = 0.05. Significance was tested between samples without Dex and with Dex treatment of the same transfection type. (b) Levels of Firefly enzyme activity normalized to* Renilla *enzyme activity measured with the luciferase reporter assay for the same transfected cells as in panel (a). Bars represent averages and confidence intervals; alpha = 0.05. Significance was tested between controls (−Dex) and treatments (+Dex), with one or two asterisks denoting *P* < 0.05 or *P* < 0.005, respectively.

EpH4 cells were cotransfected with a control reporter construct expressing constitutively the Renilla luciferase, under control of the HSV thymidine kinase promoter (pRL-TK) to control and normalize transient transfection efficiencies. Confluent EpH4 cells were treated with Dex during and 4 hours prior to the transfection to ensure that Dex associated complexes were formed before the expression of luciferase. Protein levels of both reporter vectors (Firefly and Renilla) were measured after transfection and Matrigel addition. Although downregulation of AQP5 mRNA with Dex was verified in transfected cells, no reduction in synthesis of bioluminescent reporters was measured in the luciferase activity assay (Figure 6). On the contrary, cells treated with Dex presented slightly higher (yet, not significantly different) synthesis levels for the reporter vectors (Figure 6). The inserted AQP5 promoter was functional given that the promoter-less (empty) reporter vector (pGL3-basic) showed significantly less luciferase activity (Figure 6).

## 4. Discussion

In this work, we used the mouse mammary epithelial cell line EpH4 to examine the role of AQP5 promoter methylation in the regulation of AQP5 in the mammary gland. Our results demonstrate that Dex significantly reduced AQP5 expression, both at the mRNA and protein level in nondividing EpH4 cells, independent from changes in DNA methylation. AQP5 mRNA was downregulated a few hours earlier than protein. This delayed response might indicate that AQP5 protein levels are dependent on mRNA and also suggests that the downregulation is occurring at the level of mRNA transcription. We further explored how AQP5 transcription could be regulated by analyzing the role of nGREs, ubiquitously found in the promoter, as a potential mechanism to control AQP5 expression.

### 4.1. Downregulation of AQP5 Is Independent of Changes in DNA Methylation

How AQP5 gene transcription is modulated is not known, so we analyzed an obvious candidate: promoter methylation. Contrary to the role of promoter methylation on AQP5 expression in other cell types [11–13], where expression of AQP5 has been correlated with methylation levels of its promoter, with a reduced expression when the promoter was highly methylated, we did not observe measurable changes in methylation at different levels of AQP5 expression in EpH4 mammary gland cells. The AQP5 promoter methylation remained unchanged regardless of the drop in AQP5 expression levels, even at Sp1 sites. Yet, as expected, 5-Aza treatments resulted in a large fraction of unmethylated AQP5 promoters in actively dividing EpH4 cells, demonstrating that changes in methylation can be measured in our system. Interestingly, demethylation of the AQP5 promoter by 5-Aza did not affect the levels of AQP5 expression. We conclude that AQP5 expression is independent of the methylation status of the AQP5 promoter in EpH4 mouse mammary cells.

### 4.2. Dex Induced AQP5 Repression Is Not Regulated via nGRE

Alternatively to promoter methylation, Dex could be regulating AQP5 transcription directly by acting as a transcriptional repressor on nGREs in the promoter region. Dex is a synthetic steroid hormone of the glucocorticoid group that can control expression by transrepression, in which the glucocorticoid receptor (GR) is tethered to a transcription factor via a ligand (references within and reviewed by [27–29]). It was also observed that glucocorticoids can block transcription via an evolutionary-conserved* cis*-acting element, known as nGRE. The nGRE element mediates transrepression by binding a GC-agonist tethered to a glucocorticoid, which assembles into a repressing complex. The nGRE is characterized by two inverted repeats separated by either one or two bp (CTCC, spacer of 1-2 bp, GGAGA) that bind two GR monomers [26]. Using a luciferase reporter, it was shown that mouse and human genes with an nGRE in their promoter can be repressed by a glucocorticoid* in vitro* [26]. Moreover, genes with an nGRE in the promoter region have been shown to be transcriptionally repressed by Dex in mouse epidermis, intestinal epithelial cells, liver or human A549 lung epithelial carcinoma cells. Although AQP5 was not among the Dex-responsive genes identified in the microarray analysis [26, 30], it was recently reported that antiasthmatic agents such as Dex, ambroxol, and terbutaline reduced the mRNA and protein expression of AQP5 in the lungs of mice with acute asthma [31]. However, Dex and ambroxol had the opposite effect in human lung cells and upregulated AQP5 expression [32].

We can also conclude from our transfection experiments that the transcriptional repression of AQP5 by Dex does not directly occur on the promoter in* cis*, at least not within ~2 kb promoter sequence upstream from the translational initiation site. Our bioinformatic analysis of the 7 kb region upstream and 4.5 kb downstream of the AQP5 translation initiation site showed two bona fide nGREs with 1 bp spacer (*N* = 1) at position −3524 and in intron 4 and one nGRE without a spacer (*N* = 0) at position −1962 (Figure 5). Fourteen more nGRE elements were found also within the region, if considering one mismatch in the nGRE motifs with no spacer, one or two bp spacers (*N* = 0, 1 and 2), of which 10 had tolerable changes for repressing activity [26]. Of particular importance due to its proximity to the translation initiation site could be the nGRE motif at position −438 (Figure 5 and Supplementary Figure 7). Yet, this nGRE (*N* = 1) with the sequence C**G**CCaGGAGA (mismatch in bold) has an intolerable mismatch that does not respond to the downregulation by GC, as shown previously [26]. We tested if the second −1962 nGRE may drive the downregulation in a pGL3-based luciferase reporter containing the AQP5 murine promoter sequence with both the −438 nGRE and −1962 nGRE. Since no response to Dex was observed in the luciferase reporter, we conclude that the nGRE (*N* = 0) at position −1962 is not sufficient to drive the downregulation of AQP5, which is congruent with previous observations that nGRE (*N* = 1) and nGRE (*N* = 2) decrease expression better than nGRE (*N* = 0) [25]. The next nGRE in the AQP5 promoter region with stronger repressing activity is at position −3524 and six more nGREs are found between positions −4749 to −7000. Interestingly, 30% of the nGRE motifs in the murine AQP5 region with strong repressor activity [26] are intragenic. Whether the intragenic nGREs or the distant upstream nGREs (more than 3500 bp away from the translation initiation site) act as negative effectors of Dex, enabling binding with the GC repressing complex, remains to be seen. The analysis of the human AQP5 region also shows several nGRE elements distributed upstream, in the 5′ UTR and in intronic sites (Supplementary Figure 10). At least 3 nGREs within ~1700 bp of the translation initiation site are strong nGRE effectors [26] and could act as regulators of Dex activity in humans.

### 4.3. Proliferating Cells Have a Reduced Dex Response

Our results suggest an additional level of control related to the proliferative state of the cells. We clearly show that Dex represses AQP5 strongly in nondividing cells, induced either by high cell density (confluent cells) or by Matrigel treatments. In actively dividing EpH4 cells, the reduction of AQP5 mRNA expression by Dex was attenuated, if not absent. This differential response might be related to the differentiation state of the cells. Cells form mammospheres and produce milk proteins when cultured with Matrigel and a lactogenic mixture of Dex, insulin, and prolactin [15]. Mammospheres are considered a morphological criterion for differentiation of EpH4 cells, which includes the polarization of cells into a basal and apical orientation [15, 33]. In our experimental system, we also observed the formation of mammospheres when culturing EpH4 cells with Dex and either with Matrigel or >90% confluence (empirical observations). Thus, it is possible that nondividing, contiguous cells have already progressed further into differentiation compared to dividing cells and have therefore a different response to Dex. The reduction of AQP5 in nondividing cells observed* in vitro* is consistent with* in vivo* reports, where AQP5 expression is reduced in mammalian epithelial cells during the maturation of the mammary gland during pregnancy and lactation [3, 4].

Whether AQP5 reduction is causal for morphological changes, or vice versa, is not fully understood, but, in an earlier report, high levels of AQP5 were measured in biopsies of invasive ductal carcinoma of human breast, in which ductal epithelial cells had lost apical polarity [7]. Moreover, Jung et al. showed that cell proliferation and migration (a change in the cellular state) of the human breast cancer cell line MCF-7 could be directly affected when reducing AQP5 by osmotic stress or an inhibitory RNA [7]. Interestingly, when we analyzed the MCF-7 cell line, we observed that this cell line had much lower AQP5 expression levels than EpH4 cells (Supplementary Figure 11).

## 5. Conclusions

In summary, our results show that Dex reduces AQP5 levels in epithelial mammary mouse cells in nondividing cells. The fact that mRNA was reduced before protein levels suggests a regulation at the transcriptional level; yet, obvious candidates for this transcriptional regulation were not identified. Although AQP5 expression has been correlated with methylation levels of its promoter in other tissues, we demonstrated that AQP5 promoter methylation was not correlated with mammary gland AQP5 expression; thus, AQP5 promoter methylation is not a suitable target of new therapeutic agents for regulating mammary gland AQP5.

Given the strong effect of the cell density or the cell proliferative activity in the Dex induced regulation of AQP5, it is likely that this regulation does not depend on binding to promoter nGRE but is influenced indirectly by mechanisms present in more differentiated cells. How AQP5 expression is controlled during different proliferative stages of the cell remains to be seen but is an important question, since it has potential implications in understanding how AQP5 is regulated in oncogenic expansions of breast cancer.

## Supplementary Material

The Supplementary Materials include Supplementary Figures 1-11 and Supplementary Tables 1-2:
Supplementary Figure 1: Viability assay of EpH4 cells testing different 5-Aza concentrations.Supplementary Figure 2: APQ5 mRNA and protein expression with different Dexamethasone (Dex) treatments.Supplementary Figure 3: AQP5 expression in different cell culturing systems.Supplementary Figure 4: Fluorescence intensity distribution of FACS measurements.Supplementary Figure 5: Different methods for analysis of protein expression.Supplementary Figure 6: Effect of Dex in dividing cells.Supplementary Figure 7: Sequence analysis of AQP5 promoter region.Supplementary Figure 8: Restriction analysis for assessing DNA methylation.Supplementary Figure 9: Effect of the global demethylation agent 5-Aza (5μM) in polarized, non-dividing cells.Supplementary Figure 10: nGRE motifs in the human AQP5 promoter region.Supplementary Figure 11: AQP5 expression in MCF-7 cells.Supplementary Table 1: Primers and probes.Supplementary Table 2: Bead Emulsion Amplification.

## Figures and Tables

**Table 1:** Regulation of AQP5 expression by Dex in different cell culturing systems.

	Effect of Dex	
	Fold change of AQP5 mRNA	Fold change of AQP5 protein
Dividing cells		
<70% confluent	↓ 0–2x	↓ 0–2x
Nondividing cells		
90–100% confluent	↓ 4–6x	↓ 4–6x
Matrigel treated cells	↓ 4–6x; after 2 h	↓ 4–6x; after 6 h
