# Supplementary material for: Aquaporin 5 Expression in Mouse Mammary Gland Cells Is Not Driven by Promoter Methylation
Source: Biomed Res Int. 2015 Feb 12;2015:460598. doi: 10.1155/2015/460598 (PMC4342075; doi:10.1155/2015/460598)
Supplement: Supplementary file 1 — The Supplementary Materials include Supplementary Figures 1-11 and Supplementary Tables 1-2: Supplementary Figure 1: Viability assay of EpH4 cells testing different 5-Aza concentrations.Supplementary Figure 2: APQ5 mRNA and protein expression with different Dexamethasone (Dex) treatments.Supplementary Figure 3: AQP5 expression in different cell culturing systems.Supplementary Figure 4: Fluorescence intensity distribution of FACS measurements.Supplementary Figure 5: Different methods for analysis of protein expression.Supplementary Figure 6: Effect of Dex in dividing cells.Supplementary Figure 7: Sequence analysis of AQP5 promoter region.Supplementary Figure 8: Restriction analysis for assessing DNA methylation.Supplementary Figure 9: Effect of the global demethylation agent 5-Aza (5μM) in polarized, non-dividing cells.Supplementary Figure 10: nGRE motifs in the human AQP5 promoter region.Supplementary Figure 11: AQP5 expression in MCF-7 cells.Supplementary Table 1: Primers and probes.Supplementary Table 2: Bead Emulsion Amplification. [file 460598.f1.pdf]

Supplementary Materials

Supplementary Figures

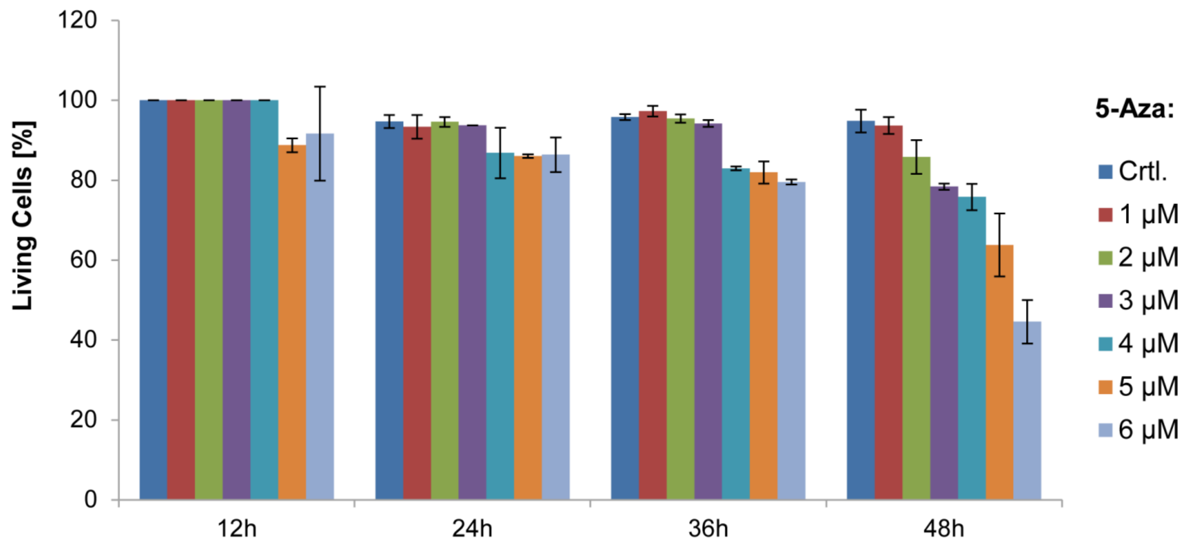

Supplementary Figure 1. Viability assay of EpH4 cells testing different 5-Aza concentrations. Dividing cells were treated with 1, 2, 3, 4, 5 and 6 µM 5-Aza to get an optimal concentration where cell viability is not affected. Cell viability was tested after 12, 24, 36 and 48h of treatment. Cells were trypsinized, washed with PBS and diluted 1:1 with 0.4% Trypan blue solution (Sigma). After 5 min of incubation, the numbers of living and dead cells were counted using a Bright-Line Hemacytometer (Sigma) and the total cell number was calculated using the Neubauer ruling.

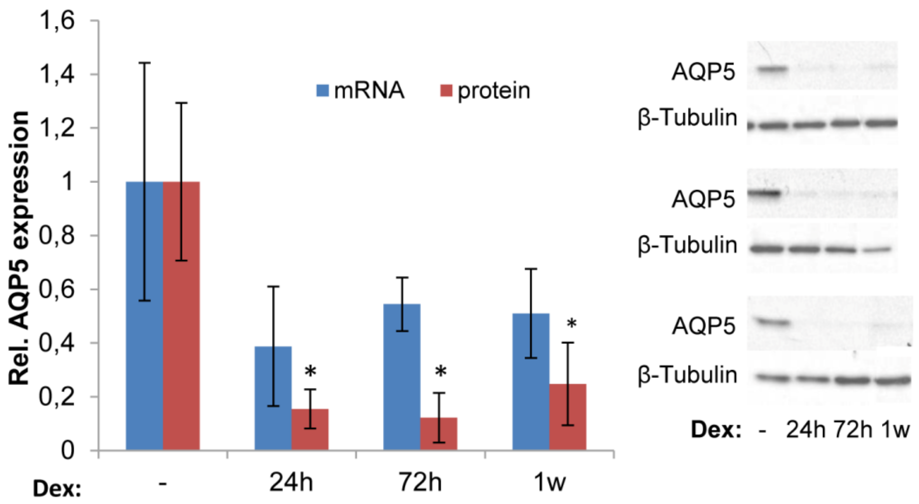

Supplementary Figure 2. APQ5 mRNA and protein expression with different Dexamethasone (Dex) treatments. AQP5 mRNA and protein were extracted from the same cell pools. Changes in mRNA expression as a result of Dex treatment were analyzed with qPCR (blue) and changes at the protein level by Western blot (red). Cells were treated with Dex for 24h, 72h and 1w. The expression of mRNA is shown relative to GAPDH, protein expression is shown relative to TUBB. Bars represent averages and confidence intervals; n=3; alpha=0.05. Significance was tested between control (-Dex) and differently treated samples.

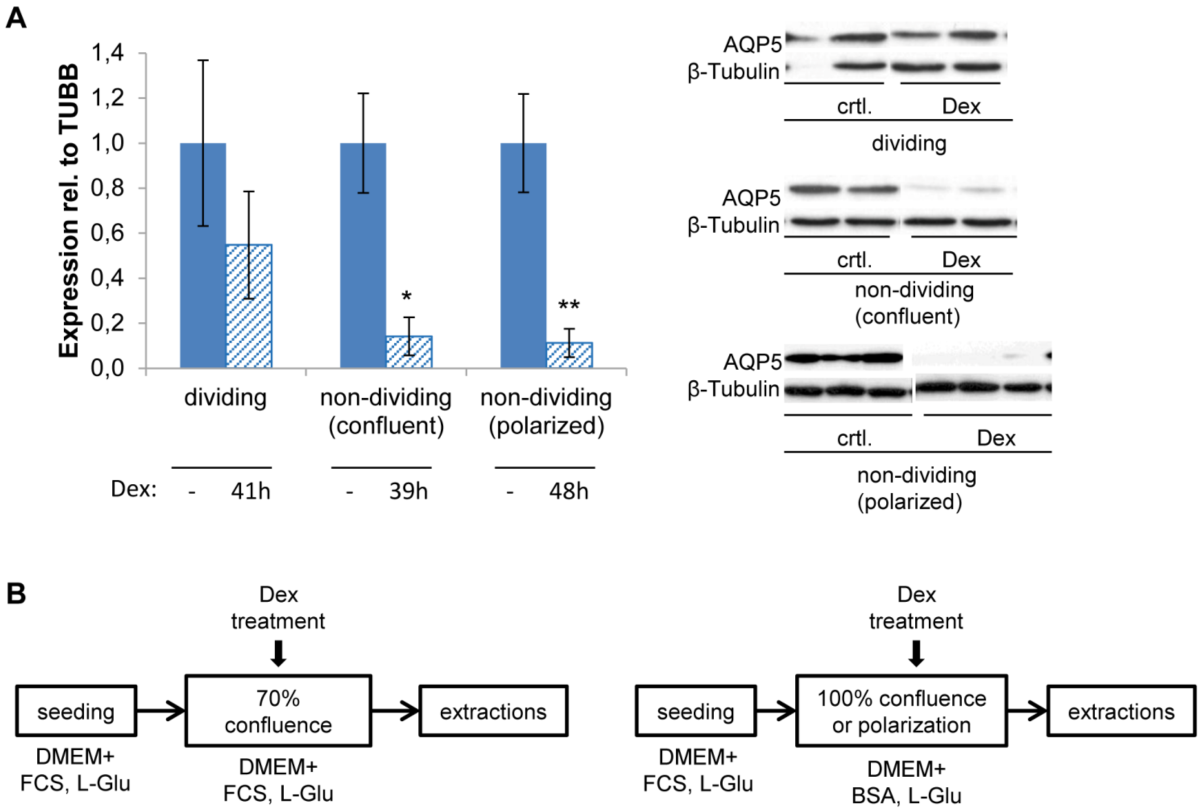

Supplementary Figure 3. AQP5 expression in different cell culturing systems. (A) Dex was added to dividing cells, non-dividing confluent cells, and non-dividing, polarized cells treated with Matrigel. AQP5 protein levels were measured by Western blot. Data were normalized to tubulin and shown relative to untreated samples. Bars represent averages and confidence intervals; n=2 in case of dividing and non-dividing, confluent conditions, and n=3 for non-dividing, polarized conditions; α=0.05. Significance was tested for each cell culturing condition between controls (-Dex) and Dex treated samples. The Dex treatment length is shown for each culturing system. **(B)** Overview of cell culturing conditions of dividing, non-dividing (confluent), and non-dividing (polarized) cells.

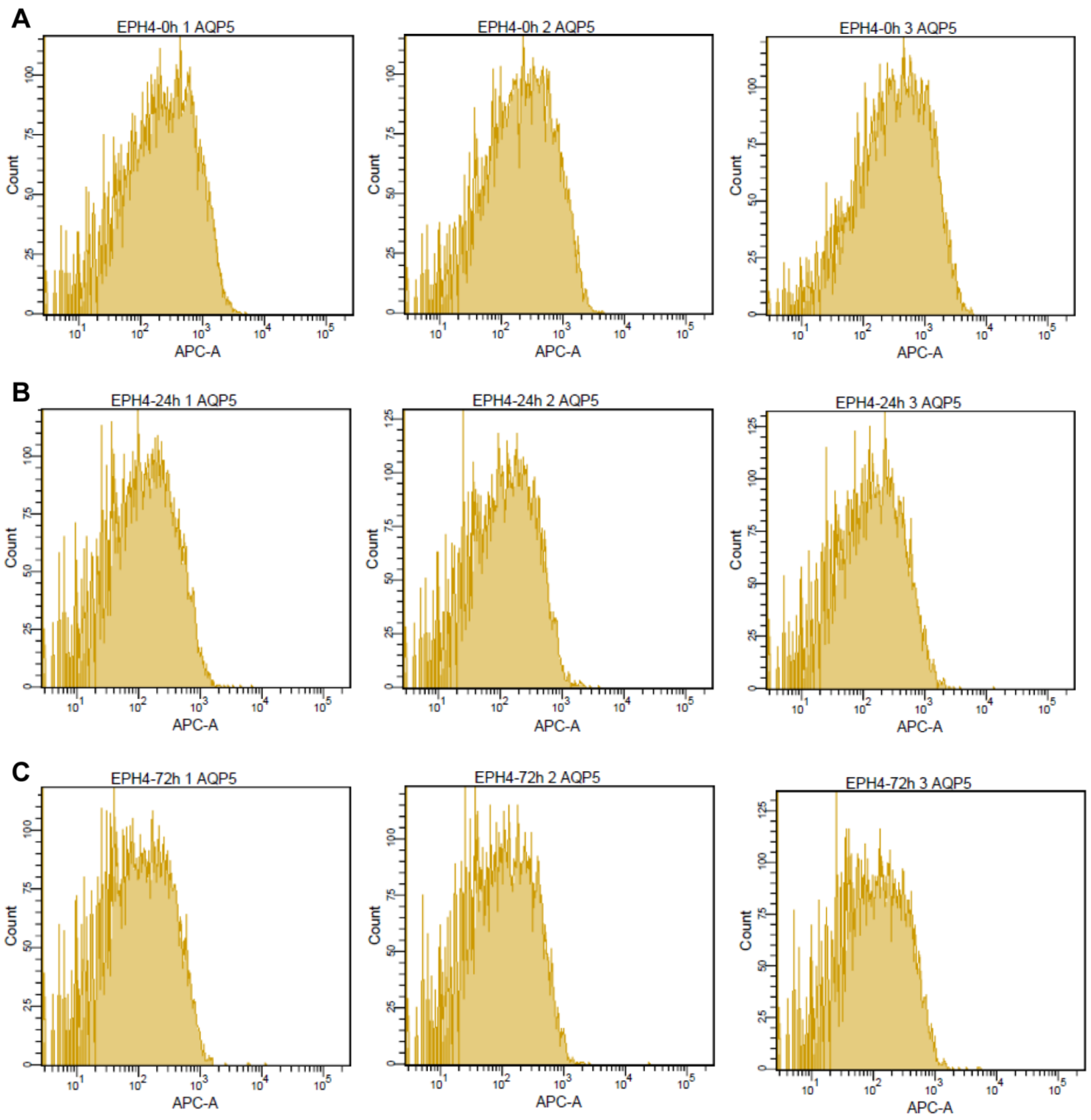

Supplementary Figure 4. Fluorescence intensity distribution of FACS measurements. Fluorescence signal intensities measured for the secondary Cy5 conjugated antibody (APC-A) plotted against the number of counted cells (Counts). **(A)** Intensity distribution for the three biological replicates of the untreated control (without Dex). **(B)** Cells treated with Dex for 24h, **(C)** cells treated with Dex for 72h.

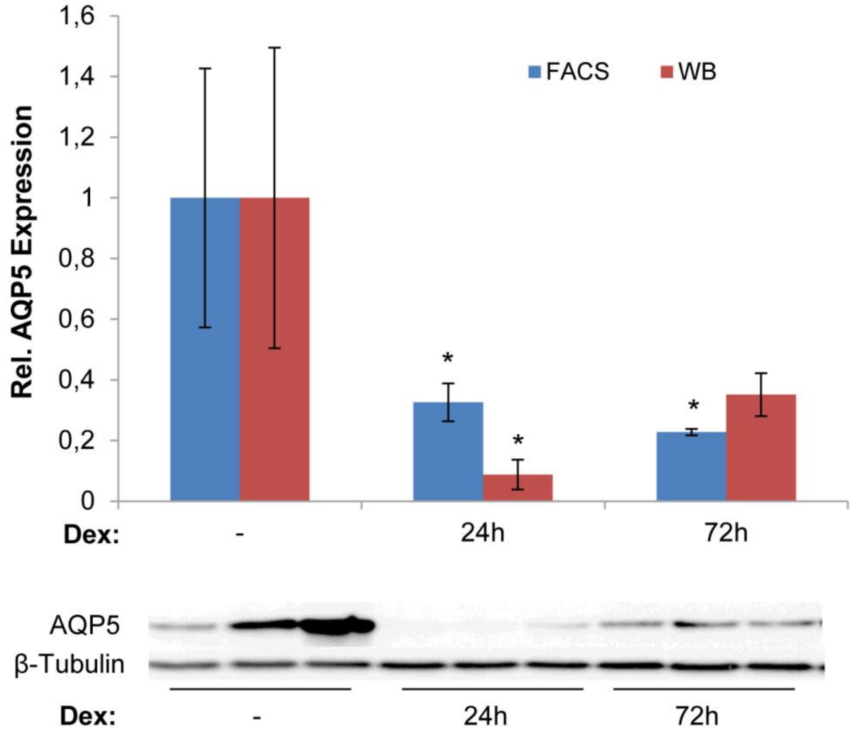

Supplementary Figure 5. Different methods for analysis of protein expression. Changes in AQP5 expression were investigated with two different Methods: FACS (blue) and Western blot (red). FACS data is shown relative to an isotype control (IgG), which was subtracted. In Western blot data, AQP5 expression is shown relative to TUBB and shown relative to untreated samples. Bars represent averages and confidence intervals; n=3; alpha=0.05.Significance was tested between control (-Dex) and differently treated samples.

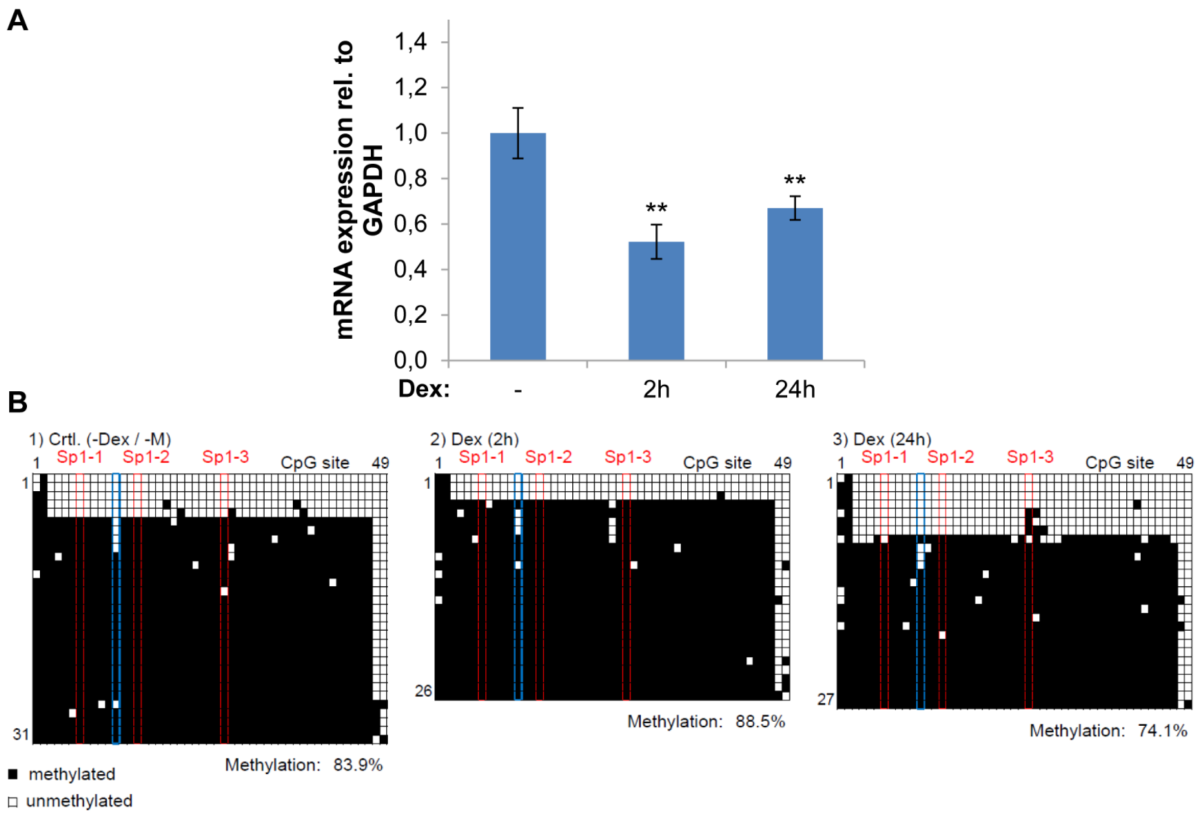

Supplementary Figure 6. Effect of Dex in dividing cells. Cells were subjected to Dex treatment for 2h and 24h prior to extraction. Cells were harvested at about 70% confluence. **(A)** Changes in AQP5 mRNA expression were analyzed with one-step qPCR and normalized to GAPDH. Columns represent averages with confidence intervals; n=12; α=0.05.Significance was tested between control (-Dex) and differently treated samples. **(B)** DNA methylation of the AQP5 promoter region measured by bisulfite sequencing of individual clones derived from DNA pools of 12 replicates.

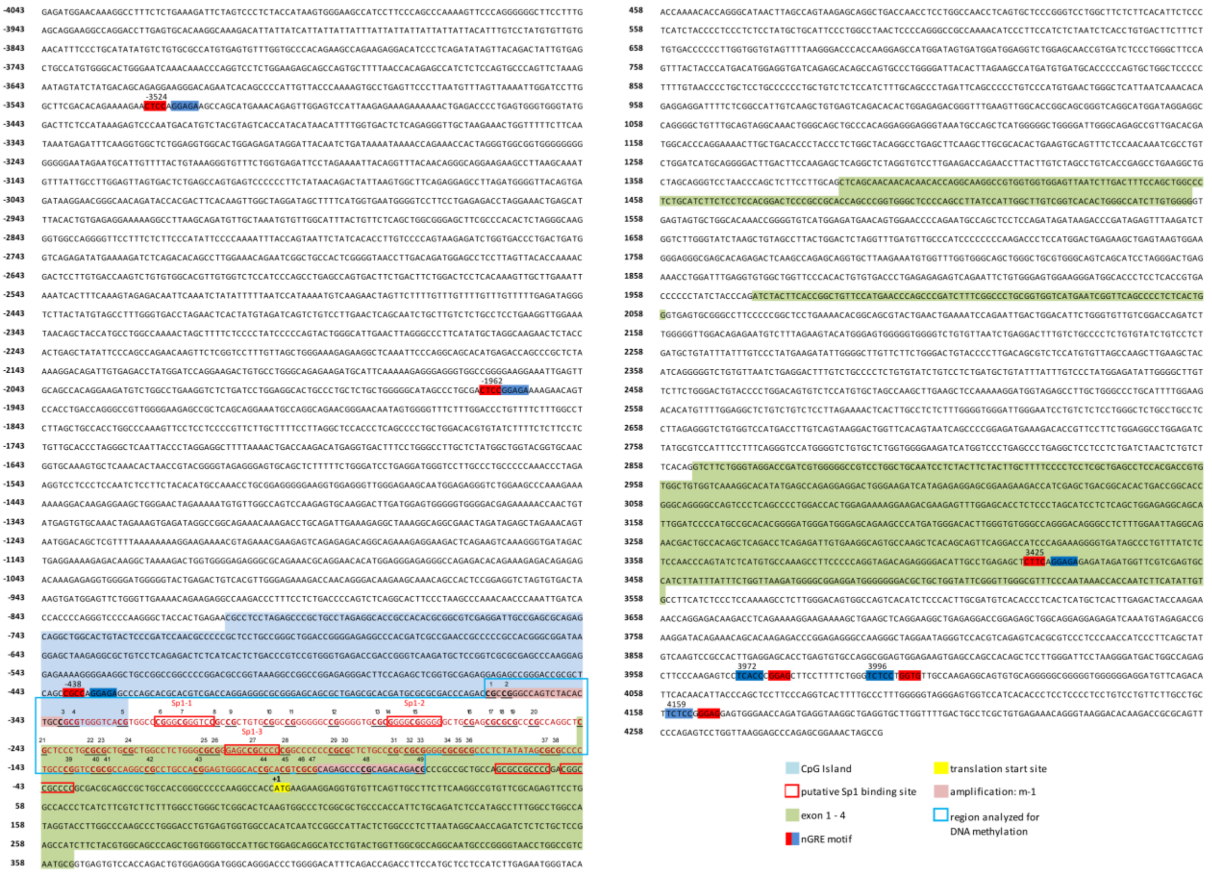

Supplementary Figure 7. Sequence analysis of AQP5 promoter region. Sequence positions are calculated relative to the translation initiation site. Position -4043 equals the chromosomal position chr15: 99587229 (GRCm38/mm10). A putative CpG island is highlighted in blue reaching from position -809 to 372. The region amplified with the primer m-1 is shown in red font, primer sequences are listed in **Supplementary Table 1**. Five identified putative Sp-1 binding sites are framed in red. Three of them are located within the region analyzed by sequencing (-73 to -361; framed in blue). CpG sites within this region are numbered from 1-49. CpG #24 represents the site analyzed using BEA. Identified nGRE motifs are marked in red/blue boxes.

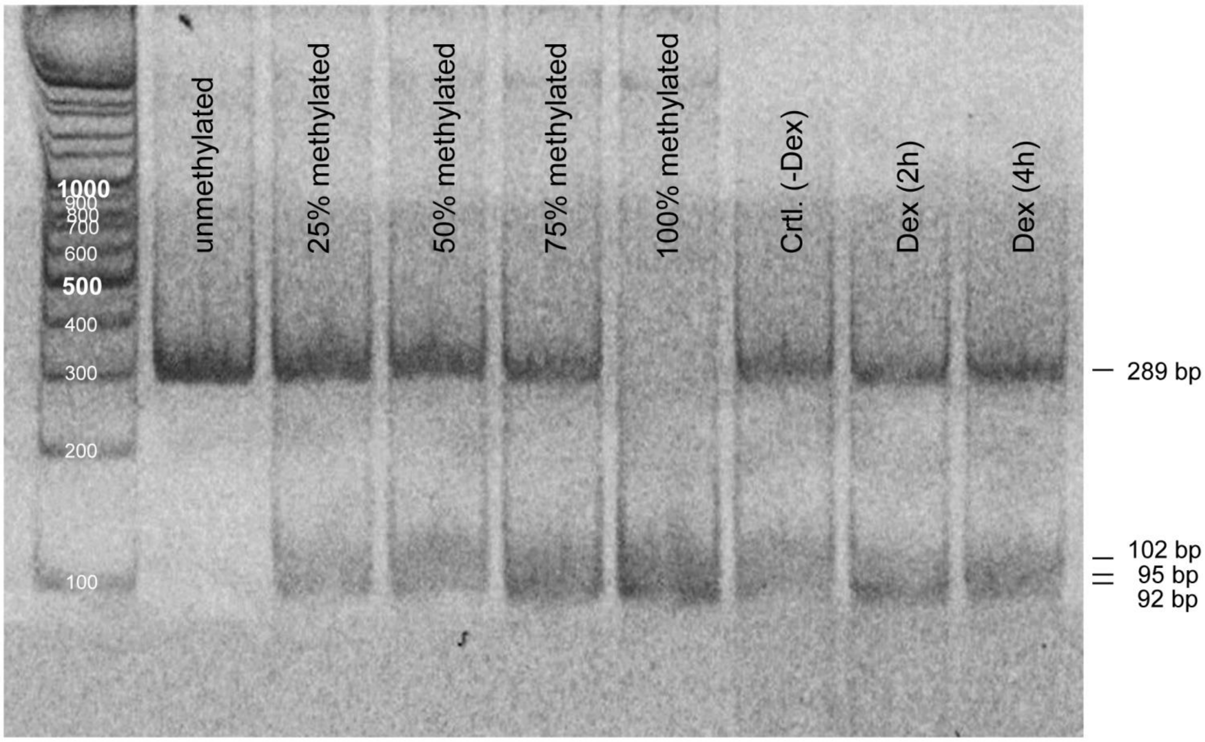

Supplementary Figure 8. Restriction analysis for assessing DNA methylation. Restriction patterns of differently methylated standards (0, 25, 50, 75 and 100% methylated) digested with BssHII. Also shown are digests of samples treated with Dex for 2h and 4h or without Dex.

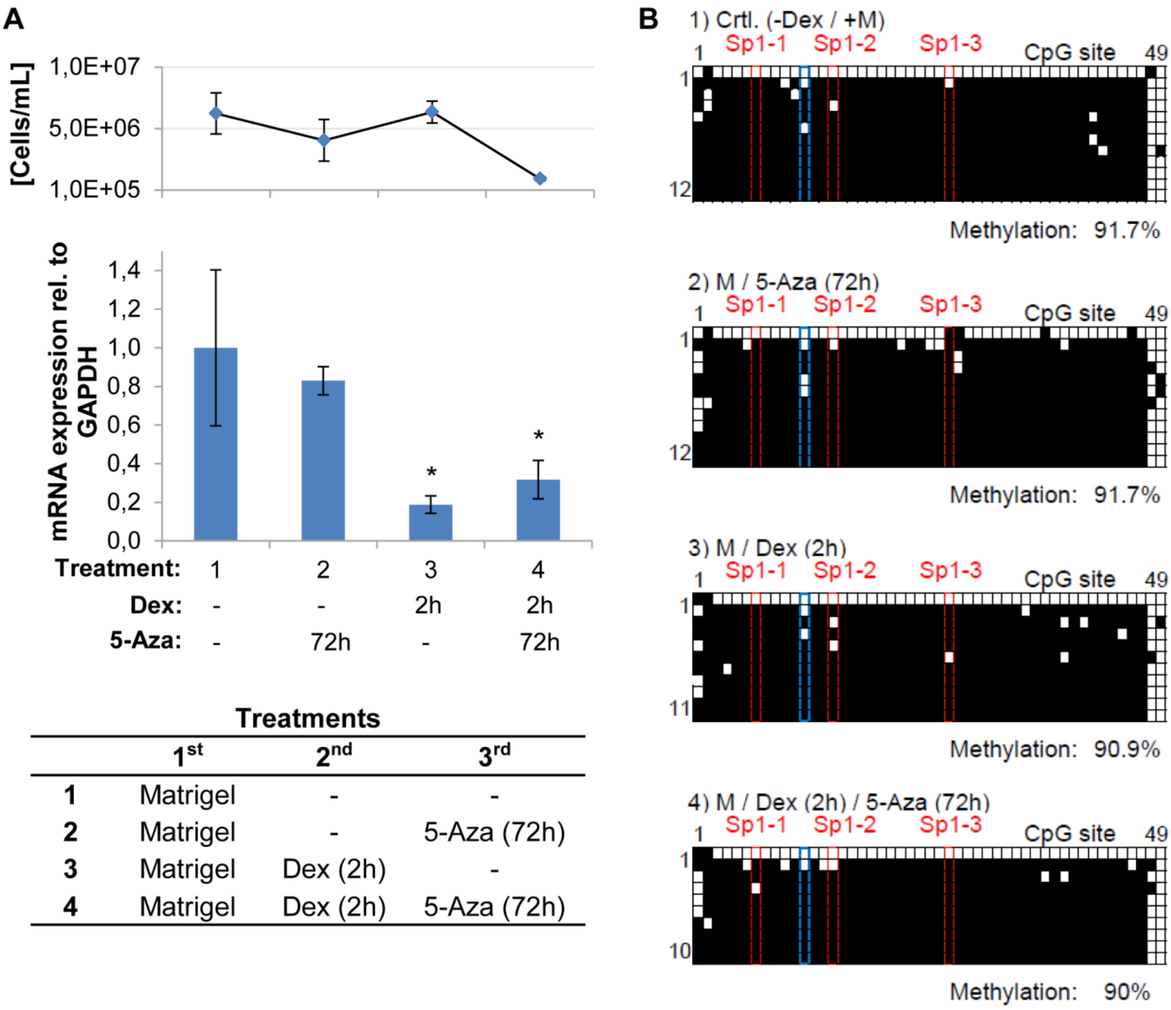

Treatments

	1st	2nd	3rd
**1**	Matrigel	-	-
**2**	Matrigel	-	5-Aza (72h)
**3**	Matrigel	Dex (2h)	-
**4**	Matrigel	Dex (2h)	5-Aza (72h)

Supplementary Figure 9. Effect of the global demethylation agent 5-Aza (5µM) in polarized, non-dividing cells. (A) Shown are numbers of cultured cells counted with a Coulter Counter and changes in AQP5 mRNA expression analyzed with one-step qPCR. Cells were treated with 5-Aza for 72h, with Dex for 2h or a combination of both treatments, as outlined in the figure table. AQP5 expression is normalized to GAPDH. Bars represent averages and confidence intervals; n=3; alpha=0.05. Significance was tested between control (-Dex, 5-Aza) and differently treated samples. **(B)** DNA methylation measured by bisulfite sequencing of individual clones (black numbers) for the same treatment conditions analyzed with qPCR.

	Sequence	Position	Context
	w/o mismatch		
N=2	CTCCCTGGAGA	2181	Intron 3
	1 mismatch		
N=0	CTCCGG**G**GA	-904 t	5‘
N=1	CTC**T**CGGAGA	-1274 t	5‘
N=2	**A**TCCCGGGAGA	*-1756 t	5‘
	C**A**CCGAGGAGA	-450 t	5‘ UTR
	CTCCATGG**G**GA	792 t	Intron 1
	CTCCAGGGA**C**A	*1386 i	Intron 1
	CTCCAGGGAG**C**	*2180 t	Intron 3
	CT**T**CAGGGAGA	*2842 t	Intron 3

Supplementary Figure 10. nGRE motifs in the human AQP5 promoter region. A ~3 kb region of the human AQP5 promoter and the AQP5 gene were analyzed for nGRE motifs. Sequence positions are calculated relative to the translation initiation site. 1 nGRE motif without a mismatch was found with 2 N as spacer. 8 nGRE motifs with 1 mismatch could be identified that contain 0, 1 or 2 N as spacer. For the motifs containing a mismatch, the mismatch was analyzed if it is tolerable (t) or intolerable (i) according to Surjit et al. [16]. Mismatches are indicated bold and underlined.

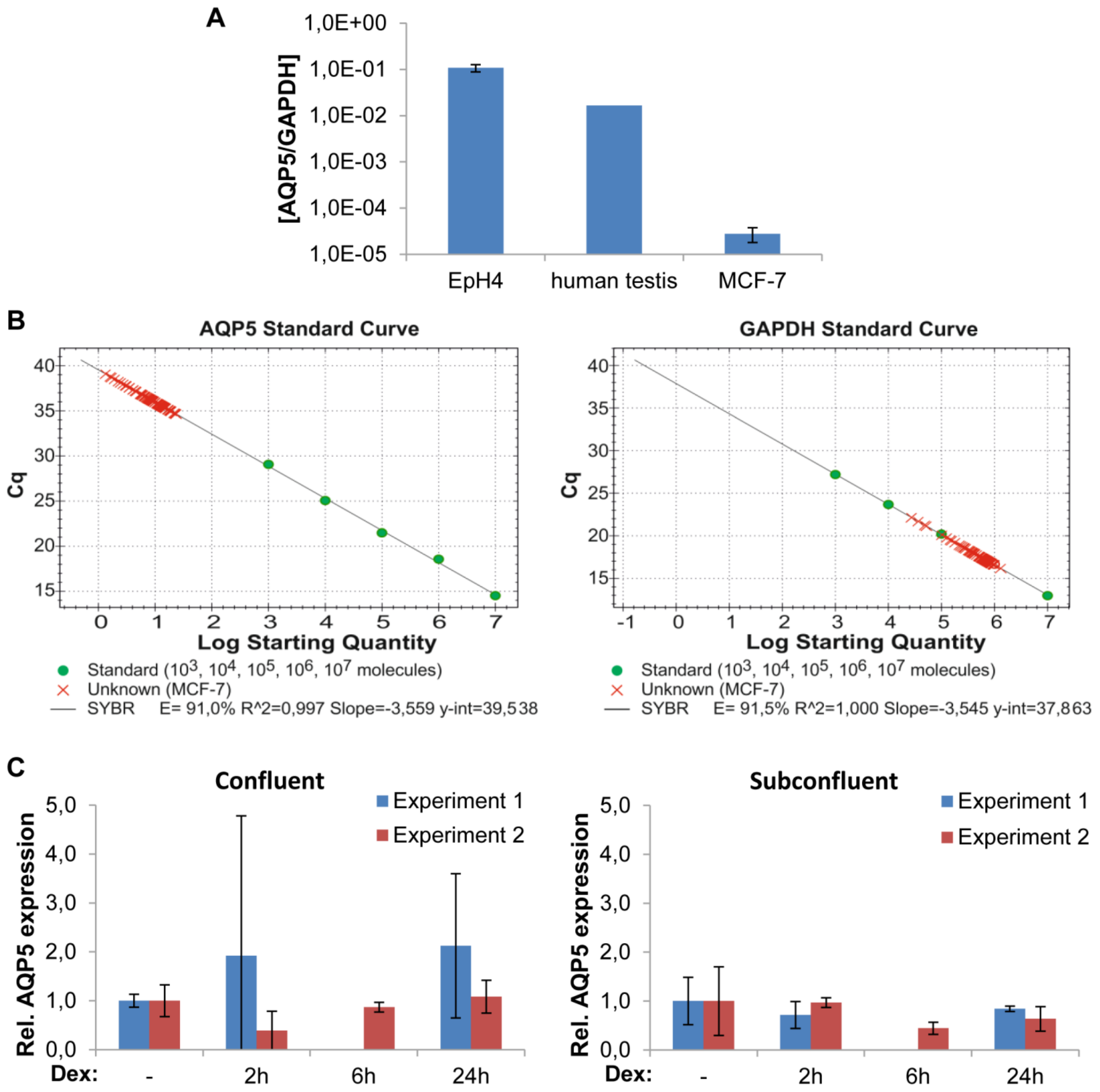

Supplementary Figure 11. AQP5 expression in MCF-7 cells. (A) Comparison of average AQP5 mRNA expression relative to GAPDH expression (obtained by qPCR) between murine EpH4 cells, a human testis biopsy, and human MCF-7 cells shows that in MCF-7 cells, AQP5 expression is very low (AQP5/GADPH ratio is ~4000 times lower compared to EpH4 cells). AQP5 mRNA expression in human testis was used as a positive control, in which differences between AQP5 and GAPDH were relatively similar to AQP5 expression in EpH4 cells (AQP5/GADPH is ~10 fold). **(B)** Standard curves obtained by qPCR are shown for AQP5 (left) and GAPDH (right) in which 10^7, 10^6, 10^5, 10^4, and 10^3 molecules of plasmid were used as standards. While GAPDH is expressed at high levels in MCF-7 cells, AQP5 expression is very low (~100,000 fold lower than GAPDH), and based on the standards, were estimated to ~1-50 AQP5 mRNA molecules per 500 ng total RNA. **(C)** AQP5 expression with different Dex treatments (2h, 6h and 24h) was analyzed on mRNA level in confluent and subconfluent MCF-7 cells in two independent experiments. The expression of mRNA is shown relative to GAPDH. Bars represent averages and confidence intervals; n=3; alpha=0.05.

Supplementary Tables

Supplementary Table 1. Primers and probes. All Primers and probes used in different experiments are listed in the table. Red letters indicate a universal sequence used to attach the specific primers to the beads.

**Name**	**Forward primer**	**Reverse primer**	**Product length**
qAQP5	5’-CTC CCC AGC CTT ATC CAT TG-3’	5’-CCA GAA GAC CCA GTG AGA GG-3’	145 bp
qGAPDH	5’-ATT GTG GAA GGG CTC ATG AC-3’	5’-GTG GAT GCA GGG ATG ATG TT-3’	125 bp
qAQP5_human	5’- CCA GCC CTG TCC ATT GGC -3’	5’- GGC TGA ACC GAT TCA TGA CC -3’	121 bp
qGAPDH_human	5’- GTG GAA GGA CTC ATG ACC AC -3’	5’- AGG CAG GGA TGA TGT TCT GG -3’	118 bp
m-1	5’-CRC CRA ACC AAT CTA CAC TAC C-3’	5’-YGTTTGTTTGYGGGGTTTTG-3’	289 bp
uc-3	5’-AGA GCC CCG CAG ACA GAC-3’	5’-TGG AGA TCT GCA GAA TGG TG-3’	230 bp
	**BEA primer/probe sequence**		
FWD1	5’-ACA CCA AAC TAC AAA ATC ACT CAT TAC CRC CAA ACT CRC TCC CTA C-3’		
REV (CA)	5’-GAG AGA CCA AGC GGA GTA GGG CGA A GYG TTT YGY GGY GGG TAG AG-3’		
REV (TA)	5’-GAG AGA CCA AGC GGA GTA GGG TGA A GYG TTT YGY GGY GGG TAG AG-3’		
REV (CG)	5’-GAG AGA CCA AGC GGA GTA GGG CGA G GYG TTT YGY GGY GGG TAG AG-3’		
RBGN-tag	5‘-GAG AGA CCA AGC GGA GTA-3‘		
R-CSX	5’-/52-Bio//iSp9/TAT GTC TTT CTC TCA CAT AA ACA CCA AAC TAC AAA ATC ACT CAT TA-3’		
AQP5 CpG 8 (C)	5’-Alexa488/TCG CGT TTA GAG GTT AGC-3’		
AQP5 CpG 8 (T)	5’-Alexa594/TCG CGT TTA GAG GTT AGT -3’		
	**AQP5 promoter cloning**		
AQP5_Region3	5’-AGC AGG CTG GCA CTG TACTCC-3‘	5'-cgtcgtaagcttGGTGGCCTTGGGGGCC -3'	757 bp

Supplementary Table 2. Bead Emulsion Amplification. Bisulfite converted DNA from samples without Dex treatment, treated with Dex for 2h and for 4h was analyzed by BEA for DNA methylation. Shown are the total number of beads (each representing one initial template molecule) counted to be methylated or unmethylated.

**Treatment:**	**without Dex**	**Dex (2h)**	**Dex (4h)**
Methylated [#]	119347	119438	89492
Unmethylated [#]	19654	16410	16667
**Methylated [%]**	**85.86**	**87.92**	**84.30**
